# Antiphospholipid Antibody Testing in a General Population Sample from the USA: An Administrative Database Study

**DOI:** 10.1038/s41598-020-59990-5

**Published:** 2020-02-20

**Authors:** Giordano Egiziano, Jessica Widdifield, Anisur Rahman, Evelyne Vinet, Cristiano S. Moura, Jeffrey R. Curtis, Sasha Bernatsky

**Affiliations:** 1 McGill University, Division of Rheumatology, Montreal, Quebec Canada; 2 University of Toronto Institute of Health Policy, Management and Evaluation, Toronto, Ontario Canada; 3 University College London, Division of Medicine, London, UK; 4 McGill University, Division of Epidemiology, Montreal, Quebec Canada; 5 The University of Alabama at Birmingham, Division of Clinical Immunology and Rheumatology, Birmingham, Alabama USA

**Keywords:** Systemic lupus erythematosus, Risk factors

## Abstract

We sought to characterized patterns of aPL testing in a large general population sample from the United States. Using Truven Health MarketScan laboratory data from 2010–2015 we identified individuals tested for lupus anticoagulant(LA), anti-cardiolipin (aCL), and anti-beta2-glycoprotein1(aGP1). Our research was approved by the McGill institutional review board (A04-M47-12B). We identified 33,456 individuals with at least one aPL test. Among these, only 6,391 (19%) had all three tests (LA, aCL, aGP1) performed. Confirmatory aPL testing was performed at least 12 weeks later in 77%, 45%, and 41% of initially positive LA, aCL, and aGP1, respectively. Of those re-tested after ≥12 weeks, only 255 (10.6%) were found to have a confirmatory positive aPL test. These findings highlight that aPL testing may often be incompletely performed. Further investigations will be required to better understand the low rate of a confirmatory positive aPL test ≥12 weeks after the initial test.

## Introduction

The antiphospholipid syndrome (APS) is defined by vascular thrombosis or pregnancy morbidities in the presence of persistently circulating antiphospholipid antibodies (aPL)^1^. APS may arise secondarily in patients with autoimmune diseases, particularly systemic lupus erythematosus (SLE), or be a primary condition unassociated with an autoimmune disease^2,3^. There is increasing evidence that asymptomatic individuals who are positive for multiple types of aPL have the greatest thrombotic risk^4,5^. Furthermore, little is known regarding whether clinicians test for aPL according to the revised Sydney APS classification criteria^1^. Since aPL may be transiently elevated by infections, malignancy or certain medications, repeat testing at an interval of 12 or more weeks is required to confirm the presence of a circulating aPL when establishing a diagnosis of APS^2^. Given the various gaps in our current understanding of aPL testing in practice, we sought to characterize patterns of aPL testing in a large general population sample.

## Materials and Methods

The Marketscan Research Databases provides United States administrative health data contributed by large employers, managed care organizations, hospitals, electronic medical record (EMR) providers, Medicare and Medicaid. Data comprise service-level claims for inpatient and outpatient services. Individuals within the MarketScan Laboratory Database were linked to the MarketScan Commercial Claims and Encounters Database and MarketScan Medicare Supplemental and Coordination of Benefits Database to obtain detailed medicals claims for healthcare services performed in both inpatient and outpatient settings. These claims databases have been de-identified and standardized for research purposes. Analyses were confined to a subset of patients with available laboratory testing, which included approximately 6.8 million individuals. We identified patients with any aPL [lupus anticoagulant (LA), anti-cardiolipin (aCL), and anti-beta2-glycoprotein 1 (aGP1)] test between 2010–2015. Screening tests for LA included the aPTT and dRVVT tests. These individuals were then described in terms of number of tests done and their results (positive or negative). A positive aPL test was defined as a confirmatory test ratio >1 for LA, aCL titer >40 GPL units, and aGPI titer >25 GPL units, respectively. All patients were required to be at least 18 years old, having continuous eligibility in the database with both medical and pharmaceutical benefits at least 12 months before and 3 months following the first aPL test. Among patients with an initial positive test, we determined whether follow-up testing was consistent with the revised Sydney APS classification criteria). Our research was approved by the McGill institutional review board (A04-M47-12B).

### Ethical approval

This article does not contain any studies with human participants or animals performed by any of the authors.

## Results

**Figure 1:**
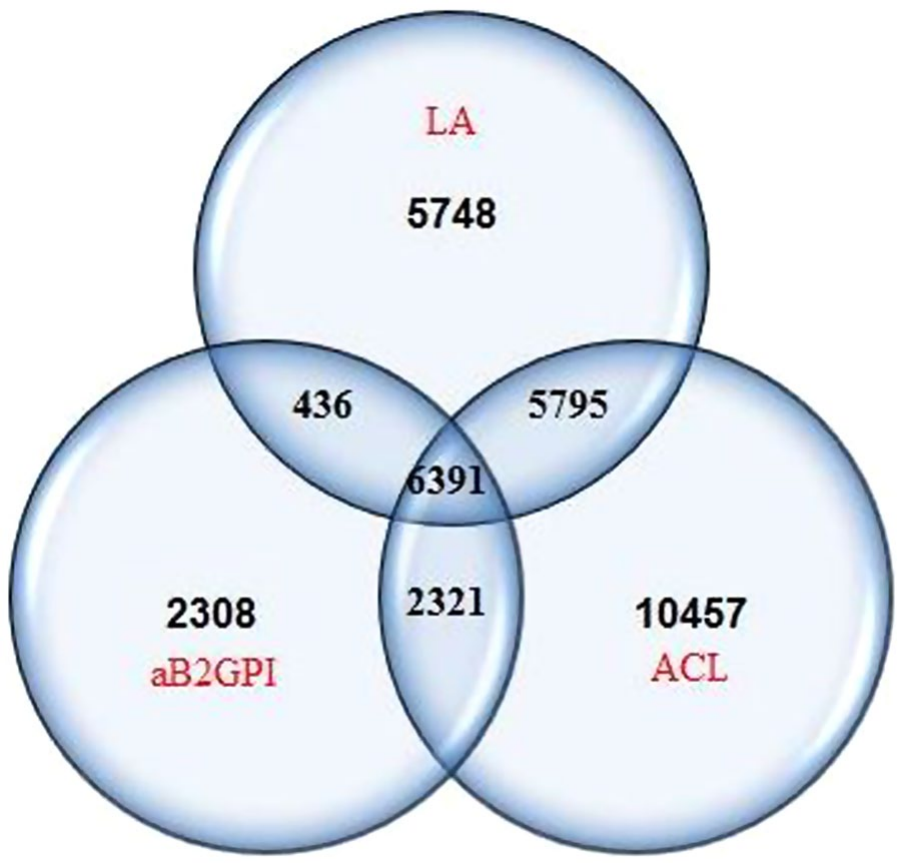
Distribution of testing for LA, aCL, and aGP.

We identified 33,456 individuals with at least one aPL test (Table 1). The distribution of testing is shown in Fig. 1. In these 33, 456 individuals, only 6,391 (19%) had been tested for all three antibodies (LA, aCL, aGP1) within the study period. Of those 33,456 tested at least once, 5,786 (17.3%) were positive for at least one aPL, among whom only 2,417 (42%) were re-tested at 12 weeks or later. Of those re-tested, 255 (10.6%) were found to have a confirmatory positive aPL test.

**Table 1:** Results of aPL testing within a general population sample.

Criteria	Antiphospholipid Antibodies, n(%)							
	Lupus Anticoagulant (LA)		Anticardiolipin Antibodies (aCL)		anti-β_2_-glycoprotein-1 antibodies (aB2GP)		Any test (LA or aCL or aGP1)	
	Performed	Positive	Performed	Positive	Performed	Positive	Performed	Positive
At least 1 test	18370	1291 (7)	24964	3753 (15)	11456	1304 (11)	33456	5786 (17)
Same test repeated ≥6 weeks apart	1205	55 (5)	2017	194 (10)	644	84 (13)	2914	316 (11)
Same test repeated ≥12 weeks apart	996	48 (5)	1707	153 (9)	537	71 (13)	2417	255 (11)

We found 18,370 individuals with one or more LA test, among whom 1,291 (7%) were positive. Of these 1,291 initially positive for LA, 996 (77%) were known to have been re-tested at least 12 weeks later, and 48 (5%) among them had a second positive test. We identified 24,964 individuals with one or more aCL test, among whom 3,753 (15%) were positive initially. Of those 3,753 initially positive, only 1,707 (45%) were re-tested at least 12 weeks later, and 153 (9%) among them had a second positive test. We found 11,456 individuals with one or more aGP1 test, among whom 1,304 (11%) were positive initially. Of those 1,304 initially positive, only 537 (41%) were re-tested at least 12 weeks later, and 71 (13%) among them had a second positive test.

## Discussion

To comply with the revised Sydney classification criteria for APS, an initially positive aPL test must be repeated after ≥12 weeks. We determined that confirmatory aPL testing was performed ≥12 weeks in only 77%, 45%, and 41% of initially positive LA, aCL, and aGP1, respectively. Furthermore, among the those re-tested after ≥12 weeks, only 255 (10.6%) were found to have a confirmatory positive aPL test. Altogether, among individuals who had been tested at least once for any aPL, only 6,391 (19%) had been tested for all three aPL. These findings highlight that testing for APS or the presence of aPL may be incompletely performed. This may impair the detection of APS and may suggest that clinicians are not optimally profiling thrombotic risk in aPL carriers.

Only few studies have looked at the natural history of aPL carriers and reported that the overall incidence of thrombotic events was between 0.65–1.8% person-year^5–7^. In a national Finnish cohort, 119 asymptomatic aPL carriers were followed for a mean of 9.1 years. The annual rate of a first thrombotic event (venous or arterial) was 0.65% in individuals who had single positive aPL, approximating the annual thrombotic risk of the general population^5^. On the other hand, asymptomatic aPL carriers who were concurrently positive for 2 or 3 aPLs had an annual thrombotic risk of 1.27%^5^. Given the relative increased risk of thrombotic events in certain aPL carriers, primary prophylaxis in these individuals has remained controversial. This issue was further studied in a recent Cochrane review where the authors found no significant benefit or risk of primary prevention of thrombosis in aPL carriers using anticoagulant prophylaxis or aspirin versus placebo or no intervention^8^. However, support remains for prophylaxis with low-dose aspirin in certain individuals with high-risk aPL profiles (including LA, double/triple aPL positivity, or persistently high aPL titers) as the incidence rate of thrombosis in this population may be as high as 5% person-year^4,7^. These findings have recently been incorporated into the 2019 EULAR recommendations for the management of APS, where low-dose aspirin is recommended in asymptomatic individuals for primary prophylaxis when there is evidence of persistent high-risk aPL profiles^9^.

We are unable to comment on whether aPL testing within this general population sample suggested over-use or under-use of tests per se. As we have described, only 17% of individuals tested had an initial positive aPL, and among those only 10% had a second positive ≥12 weeks later. Unfortunately, we were not able to further stratify our data (ie: indication, comorbidities, medications, thrombosis history or requesting physician specialty) to assess for testing patterns within specific at-risk patient populations. Although the revised Sydney criteria have good sensitivity and specificity for diagnosing APS in individuals with known rheumatic conditions, it is known that these criteria perform less well in a general population^1,10^. Additional studies including relevant clinical risk factors will be needed to further investigate the low rate of positive confirmatory aPL tests.

As a retrospective observational study, our results are subject to inherent limitations of this study design. We have not characterized clinical factors in the patients undergoing aPL testing, such as underlying comorbidities, medications, thrombosis/pregnancy adverse events, or requesting physician specialty. Although we managed to identify a large sample of individuals tested for aPL over a 5 year period, it is possible that these individuals had undergone testing (either initial or confirmatory) outside of our study window. This may have occurred when individuals changed insurance companies or testing centers during the study period and were therefore no longer included in the MarketScan database. It is also likely that variability within laboratory reporting of a positive aPL test, as well as underlying patient anticoagulation may have influenced the decision to re-test ≥12 weeks. Finally, we were unable to provide demographic data for our results. The generalizability of our findings should be considered within the scope of MarketScan, which includes insured employees and their dependents, which typically include active employees, early retirees, Consolidated Omnibus Budget Reconciliation Act coverage and Medicare-eligible retirees with employer-provided insurance^11^.

In summary, we have reported that in individuals with a positive aPL test, confirmatory testing was performed ≥12 weeks in 77%, 45%, and 41% of initially positive LA, aCL, and aGP1, respectively. In addition, in those individuals who had been tested at least once for any aPL, only 6,391 (19%) had been tested for all three aPL. Finally, we have also reported a low rate (10.6%) of positive confirmatory tests after ≥12 weeks among individuals with an initial positive aPL test. These findings raise the possibility that aPL testing in a general population may be incompletely performed. On one hand, clinicians may be globally ordering too many screening tests for aPL in the general population, while on the other hand not including all three aPL tests (LA, aCL and aGP1). Further characterization of testing patterns in individuals with known thrombotic events, pregnancy morbidity, SLE, and other high-risk conditions is warranted.
